# Editorial: Case reports in neuroimaging and stimulation

**DOI:** 10.3389/fpsyt.2023.1264669

**Published:** 2023-08-02

**Authors:** Simone Battaglia, André Schmidt, Stefanie Hassel, Masaru Tanaka

**Affiliations:** ^1^Center for Studies and Research in Cognitive Neuroscience, Department of Psychology “Renzo Canestrari”, Alma Mater Studiorum Università di Bologna, Cesena, Italy; ^2^Department of Psychiatry (UPK), University of Basel, Basel, Switzerland; ^3^Department of Psychiatry, Mathison Centre for Mental Health Research and Education, University of Calgary, Calgary, AB, Canada; ^4^ELKH-SZTE Neuroscience Research Group, Danube Neuroscience Research Laboratory, Eötvös Loránd Research Network, University of Szeged (ELKH-SZTE), Szeged, Hungary

**Keywords:** neuroimaging, case reports, electric stimulation therapy, depression, comorbidity, Alzheimer's disease, Parkinson's disease, bipolar disorder

The brain, a remarkable and intricate system, plays a fundamental role in shaping our behavior, encompassing cognitive and emotional processes (1–3). Understanding its structural and functional organization has been greatly enhanced through the utilization of neuroimaging and brain stimulation techniques. These powerful tools not only provide insights into the complex workings of the brain but also hold promise as potential therapeutic interventions for mental disorders (4). By leveraging these techniques, researchers gain valuable insights into the underlying mechanisms of mental disorders and their potential treatments. The combination of neuroimaging and brain stimulation holds great promise for accelerating the development of symptomatic therapies and introducing novel treatments to patients. To delve deeper into the mechanisms of neuropsychiatric disorders, researchers utilize various neuroimaging techniques, such as structural and functional magnetic resonance imaging (s/fMRI), electroencephalography (EEG), diffusion tensor imaging (DTI), positron emission tomography (PET), and single-photon emission computed tomography (SPECT), all of which offer detailed visualizations and measurements of brain structure, function, and chemistry (5). These tools are essential in several areas: identifying targets for intervention, guiding neurosurgical planning, determining optimal stimulator placement, and confirming post-operative procedure effectiveness.

Neuroimaging also assists with pre-treatment screening to identify potential responders and post-treatment evaluation to assess changes in brain circuitry associated with clinical outcomes. Researchers have used multimodal neuroimaging tools to find new neurobiological mechanisms behind neuropathogenesis, stimulation effects, brain responses, and the effectiveness of therapies. More attention has been paid to altered brain regions like the prefrontal cortex (PFC), which is involved in executive functions, emotional regulation, and making decisions. Several neuropsychiatric disorders (6–8) have been linked to this brain region. Neuroimaging studies have revealed structural and functional alterations in the PFC in conditions such as depression, schizophrenia, and addiction (9–11). Understanding these alterations and their relationship to disease mechanisms is crucial for developing targeted interventions (12–14).

Brain stimulation techniques, including invasive (e.g., deep brain stimulation) and non-invasive methods (e.g., transcranial magnetic stimulation [TMS], transcranial current stimulation [tCS]), aim to modulate neural activity in specific brain regions and circuits. Non-invasive brain stimulation techniques (NIBS) like TMS and tCS have gained attention in neuropsychiatry as they offer a safe and non-surgical approach to manipulate neural circuits involved in neuropsychiatric disorders. NIBS allows precise targeting of specific brain regions by placing the stimulation device over a particular area of the scalp. For instance, in depression treatment, stimulating the dorsolateral PFC can alleviate depressive symptoms by modulating mood regulation (15, 16). These techniques are customizable to an individual's unique characteristics, enhancing their potential in clinical practice.

Recent studies highlight distinct patterns of neural activity associated with various neuropsychiatric disorders, characterized by specific frequency bands. Personalized brain stimulation techniques utilize this knowledge to adjust stimulation parameters (e.g., frequency and intensity) and target specific frequency bands related to individual symptoms (17, 18). This personalized approach shows promise in improving treatment outcomes for patients with neuropsychiatric disorders. Combining NIBS with neuroimaging methods like fMRI and EEG helps researchers gain insights into brain stimulation's effects on neural activity and connectivity between brain regions (19, 20). These techniques have shown potential in treating neuropsychiatric disorders such as depression, obsessive-compulsive disorder, epilepsy, Alzheimer's disease (AD), and Parkinson's disease (PD).

This Research Topic is dedicated to showcasing exceptional cases of patients or individuals with unexpected or unusual diagnoses, treatment outcomes, or clinical courses. These case reports offer valuable insights into the neural underpinnings of various conditions, such as differential diagnosis, abnormal emotional processes, learning mechanisms, decision-making, and the clinical management of unique cases. They serve as essential educational tools, providing novel perspectives for neuroimaging and brain stimulation studies. Proudly presenting seven papers, this collection represents a significant advancement in the field of neuroimaging and brain stimulation, contributing to our understanding of complex neurological conditions and enhancing patient care.

Five case reports focus on depression and its association with other conditions. Finding effective treatment options for patients with treatment-resistant depression is the current challenge. Rymaszewska et al. present a treatment approach for treatment-resistant depression involving combined pharmacotherapy, psychotherapy, and various neurostimulation techniques, including deep brain stimulation of the medial forebrain bundle. Another challenge involves improving early identification and intervention for atypical and early-onset AD and discovering novel pharmacological treatment targets for both AD and depression. Liu et al. present a rare case where early-onset AD initially manifested as depression, emphasizing the importance of considering AD in depression's differential diagnosis. Chang et al. explore the application of repetitive TMS to the PFC and auditory cortex, shedding light on potential therapeutic interventions for patients with tinnitus and depression. Kim et al. present a single-case study investigating clinical and functional connectivity characteristics of antidepressant-induced mania in panic disorder. Understanding mania risk factors in panic disorder is crucial due to a 20–40% risk of inducing mania during treatment with antidepressants. The study suggests that altered amygdala-based resting-state functional connectivity could serve as a potential biomarker for identifying antidepressant-induced mania in panic disorder patients. Zakia and Iskandar highlight the challenge of diagnosing and treating co-occurring psychological symptoms and rare medical conditions, like intracranial tuberculoma, particularly in cases of peripartum-onset depressive disorder. The study presents a unique clinical scenario where a depressive disorder during the peripartum period masks the presence of a suspected intracranial tuberculoma.

Another case report discusses the association between reversible splenial lesion syndrome (RESLES) and mental disorders, particularly in bipolar II disorder. Understanding the underlying mechanisms and effective treatments for RESLES in bipolar II disorder are current challenges. Zhou et al. highlight a potential link between bipolar disorder and RESLES, particularly during hypomanic episodes. Creating efficient diagnostic and therapeutic approaches that can specifically target the structural changes in the brain is one of the current challenges in the study of PD. Nyatega et al. provide insights into the structural brain changes associated with PD by investigating gray matter, white matter, and cerebrospinal fluid abnormalities in PD using voxel-based morphometry.

The combination of neuroimaging and brain stimulation techniques in neuropsychiatry offers a synergistic advantage. Neuroimaging provides a comprehensive understanding of brain function and circuitry, shedding light on the intricate mechanisms underlying neuropsychiatric disorders. Concurrently, brain stimulation allows targeted intervention, manipulating specific brain regions or networks implicated in these disorders (21–23). Integrating neuroimaging and brain stimulation helps elucidate the neural underpinnings of neuropsychiatric disorders and optimize treatment strategies. This powerful combination accelerates the development of symptomatic therapies by identifying neural circuits involved and guiding precise therapeutic interventions (4). It holds the potential to revolutionize bioelectronic medicine, paving the way for personalized treatments and transformative advances in neuropsychiatric care (17, 18, 23).

Neuroimaging and brain stimulation are not exclusive to the clinical arena. Preclinical research utilizing animal models has explored the interplay of various factors involved in mental illnesses, such as genetic, environmental, and pharmacological manipulations, shedding light on disease phenotypes and underlying pathology (2, 24–36). These models simulate disease conditions, allowing researchers to assess symptomatology and evaluate potential interventions (37, 38). They provide valuable insights into disease mechanisms, testing of treatments, and therapeutic efficacy, as well as structural changes and imaging techniques for clinical cases (39–43). The combination of preclinical and clinical research contributes to the development of innovative therapeutics and personalized medicine (44–54).

In summary, neuroimaging and brain stimulation are powerful tools in the treatment of neuropsychiatric disorders. The integration of these techniques enables a deeper understanding of the pathophysiology of these disorders and facilitates the development of more effective therapies. By combining their strengths, researchers can potentially enhance the lives of patients by bringing new and personalized treatments to the forefront of neuropsychiatric care. We hope that this Research Topic has provided valuable insights into the neural underpinnings of differential diagnosis, abnormal emotional processes, learning mechanisms, decision-making, and the clinical management of unusual cases. We thank all the authors who contributed to this collection and the reviewers who provided valuable feedback. We look forward to future contributions that will continue to advance the field of Neuroimaging and Stimulation.

## Author contributions

SB: Conceptualization, Writing—review and editing. AS: Conceptualization, Writing—review and editing. SH: Conceptualization, Writing—review and editing. MT: Conceptualization, Writing—original draft, Writing—review and editing.
